# Individuality in the Early Number Skill Components Underlying Basic Arithmetic Skills

**DOI:** 10.3389/fpsyg.2018.01056

**Published:** 2018-07-02

**Authors:** Jonna B. Salminen, Tuire K. Koponen, Asko J. Tolvanen

**Affiliations:** ^1^Department of Education, Special Education, University of Jyväskylä, Jyväskylä, Finland; ^2^Faculty of Education and Psychology, Centre for Research on Learning and Teaching, University of Jyväskylä, Jyväskylä, Finland; ^3^Faculty of Education and Psychology, University of Jyväskylä, Jyväskylä, Finland

**Keywords:** early number skill components, arithmetic, preprimary education, latent profile analysis, poorest-performing children, low-performing children

## Abstract

Early number skills underlie success in basic arithmetic. However, very little is known about the skill profiles among children in preprimary education and how the potential profiles are related to arithmetic development. This longitudinal study of 440 Finnish children in preprimary education (mean age: 75 months) modeled latent performance-level profile groups for the early number skill components that are proposed to be key predictors of arithmetic (symbolic number comparison, mapping, and verbal counting skills). Based on three assessment time points (September, January, and May), four profile groups were found: the poorest-performing (6%), low-performing (16%), near-average-performing (33%), and high-average-performing children (45%). Although the differences between the groups were statistically significant in all three number skill components and in basic arithmetic, the poorest-performing children seemed to have serious difficulties in accessing the semantic meaning of symbolic numbers that was required in the number comparison and mapping tasks in this study. Interestingly, the tasks demanding processing between quantities and symbols also most differentiated the poorest-performing children from the low-performing children. Due to remarkable and stable individual differences in early number skill components, the findings suggest systematic support and progress monitoring practices in preeducational settings to diminish and avoid potential difficulties in arithmetic and mathematics in general.

## Introduction

Typically, as an innate ability, children are able to quickly discriminate small sets of quantities without counting (1-4; subitizing range), and they can detect which of two presented quantities is larger if the difference between them is large enough (Dehaene, 2011; see also von Aster, 2000; von Aster and Shalev, 2007). It has been proposed that this ability is critical for the development of early number skills and especially for number concept skills for which children need to learn the quantitative meaning of small number words (one, two, and three; Butterworth, 2005), and later on, to map verbal and quantitative representations to corresponding number symbols. Along these skills, children recite number words very early (Fuson, 1988; Wynn, 1990; Krajewski and Schneider, 2009) which forms a base for learning exact verbal counting list (Fuson, 2009) and for enumerating and calculating quantities above the aforementioned subitizing range. To enumerate quantities correctly, children need to master and follow the procedural principles for counting (Gelman and Gallistel, 1978; one-to-one correspondence, stable order of the counting words, and cardinality). Children also need to understand what can be counted and that the order in which the quantities are counted does not matter (Gelman and Gallistel, 1978). These principles are vital for exact object counting (see also Krajewski and Schneider, 2009; Dehaene, 2011), which, in turn, relates to the development of number concept skills. Thus, understanding the association between different numerical representations that are number words, quantities, and Arabic number symbols plays a critical role in the development of early number skills (Krajewski and Schneider, 2009; Geary, 2013). Furthermore, this skill allows and strengthens the understanding of explicit number system (knowing the exact relationships between numbers) that can be seen as prerequisite for the ability to compose and decompose magnitudes and for learning efficient and flexible arithmetical calculation strategies (Krajewski and Schneider, 2009; Geary, 2013).

Atypicalities in number skills development and lack of early numerical experiences, as well as math language, increase the risk of facing challenges in learning arithmetic and mathematics at school. One main feature in mathematical learning difficulties (MD) is dysfluency in calculation skills that is deficit in arithmetic fact retrieval (Geary, 2011). That is why researchers try to draw a theoretical picture of number skills development and specify the critical early components related to arithmetic. It has been proposed that the strongest predictor of fluent arithmetic may be symbolic number processing skills (Bartelet et al., 2014; Skagerlund and Träff, 2014; De Smedt, 2015; Vanbinst et al., 2015). Children with MD might have deficits in accessing the numerical meaning from Arabic number symbols (assessed typically by number comparison task; *which of the two number symbols is larger*) which could then be related to basic arithmetic skills and math achievement in general (Rousselle and Noël, 2007; De Smedt and Gilmore, 2011; De Smedt, 2015). On the other hand, deficit in symbolic number processing might become visible in mapping task where fluent ability to transcode between non-symbolic and symbolic numerical notations is required. Previous research has shown that symbolic number comparison and mapping are separable although correlated skills, and mapping is related to mathematics achievement over and above numerical magnitude comparison skills (Brankaer et al., 2014). Deficits in mapping could also explain difficulties in understanding number relations (Geary, 2013). Finally, it has also been proposed that verbal counting plays an important role as a predictor of fluent arithmetic (e.g., Aunola et al., 2004; Zhang et al., 2014; Koponen et al., 2016), and could be a core component in identifying children with potential MD.

Before formal schooling, children typically use counting based strategies for solving simple sums and ease their counting by using manipulatives, fingers, and/or verbal counting. Later, counting strategies develop (through counting all – counting on – counting on from larger number) in consequence of repetitions and routines which in turn allow children to strengthen associations between arithmetical problems and their solutions (Peters and De Smedt, 2018). That is why verbal counting might play an important and foundational role in learning arithmetic.

As known, individual differences in early number skills appear to be relatively stable and the differences widen in subsequent years (e.g., Aunola et al., 2004; Desoete and Grégoire, 2006; Murphy et al., 2007; Geary et al., 2008; Morgan et al., 2009, 2011; Wong et al., 2014). To better understand the potential qualitative differences between the poorest-performing and low-performing children (Geary, 2011), and to give targeted support for individual needs (Dowker and Sigley, 2010) we need specific knowledge of children’s skill-profiles in separate number skill components. To date, mostly two types of studies have examined these early number skill components: studies on a certain factor (Price and Wilkey, 2017; Vanbinst et al., 2018) and studies on composite scores (Jordan et al., 2006, 2007; Aunio et al., 2015). The first type tries, more or less, to deepen the knowledge of the core factors of MD but typically does not simultaneously model two or more core components at the same time. The second type tries, more or less, to understand the developmental trajectories of number skills underlying fluent arithmetic. Neither approach allows us to draw a clear picture of how these early number skill components are related and what kind of skill profiles may exist at kindergarten age before formal schooling.

To conclude, defining a clear picture of the underlying components predicting arithmetic skills is challenging due to the varying approaches, measures, sampling issues, and age levels used in previous studies (see De Smedt et al., 2013; Lyons et al., 2014; Hart et al., 2016). Thus, we need specific knowledge of the individuality in the number skill components. This question is not only theoretically interesting but also provides new information for planning and suggesting reasonable, targeted support to prevent persistent deficits and cumulative difficulties in mathematics (Butterworth et al., 2011; Geary, 2011). One way to examine the individual differences in theoretically distinct and unique contributors of basic arithmetic is to use person-oriented analysis methods (Bartelet et al., 2014; Skagerlund and Träff, 2014). This approach tries to get support and add potential new knowledge for existing theories by driving the data instead of differentiating groups of children who are clustered with certain cut-off thresholds. The present study implemented latent profile analysis method (LPA) to investigate the heterogeneity of potential early skill profiles in the three number skill components strongly underlying fluent arithmetic: symbolic number processing (NC), mapping (MS), and verbal counting skills (VC). Along these skills, non-symbolic magnitude comparison and number line acuity also predict arithmetic achievement. However, symbolic number comparison correlates more strongly with math achievement than non-symbolic number comparison (for review see Schneider et al., 2017; within kindergarteners see Sasanguie et al., 2012). In addition, instead seeing number line acuity as a direct predictor of math skills it should be seen as a factor influencing on the developmental process of both skills (e.g., Friso-van den Bos et al., 2015). The main interest of the current study was to get more evidence of the early number skill components that challenge especially the poorest- and low-performing children the most and that are measurable for practitioners in small-group conditions by paper-and-pencil tasks.

**FIGURE 1:**
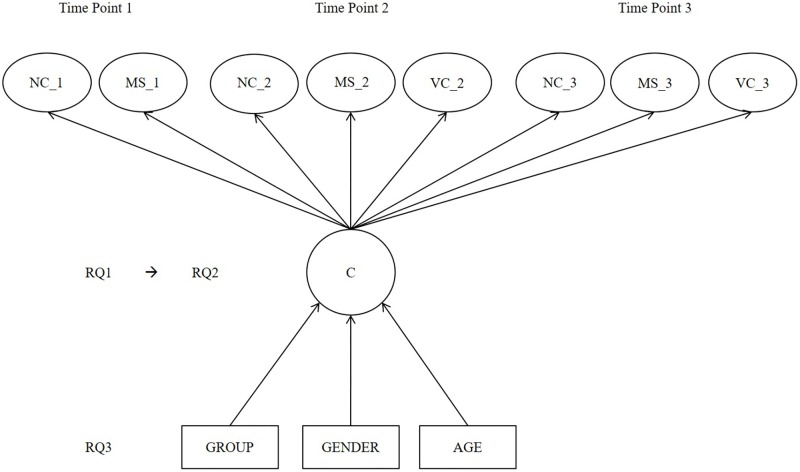
Mixture model along the three research questions of the current study. COMP(a), approximate number comparison skill; NC(c), cardinal number concept skill; VC, verbal counting skill; COMP(e), exact number comparison skill; NC(o), ordinal number concept skill; C, latent class; RQ1–RQ3, research questions 1–3.

This longitudinal study aimed first to examine whether different performance-level profile groups in early number skill components are found among children in preprimary education (research question 1, RQ1). The second aim was to examine which of the components potentially differentiates the profile groups the most (RQ2). The third aim was to examine whether the preprimary education group, gender, or age plays a role in belonging to a certain profile group (RQ3). Finally, the between-group differences in basic arithmetic were tested (RQ4). The three screening tools with negatively skewed distribution were used to assess early number skill components in September, January, and May. With this procedure, the study aimed to deepen knowledge of the skill performance of poorly performing children through the preprimary education year (for researchers) and to reliably screen children in need of extra support for numerical skills (for practitioners). Therefore, differentiation of performance levels among typical-, average-, or high-achieving children was not the focus. The theoretical model and the three main research questions are presented later in **Figure 1**.

## Materials and Methods

### Participants

At the outset, 35 kindergarten teachers voluntarily participated in the study as data collection coordinators. Parents received an information letter with the descriptions of our study purpose, procedure, and contact information. Parents were informed of their right to decline or discontinue the children’s participation to our study at any time point. Parents were also informed that we will not ask any information to identify children from the data (such as surname, birth date, etc.) and therefore, written permissions from parents to us were not required. The final sample sizes varied from 486 to 557 kindergarteners, depending on the assessment point and given the option for teachers, parents, and children to commence or cease participation at any point. Altogether, 30 teachers and 440 kindergarteners who participated in all three assessment time points were included in the analyses. These longitudinal data for Finnish kindergarteners were geographically representative, and when tested, participant attrition was not found to be systematically related to any of the early number skill components assessed in this study. The final sample consisted of 223 girls (*mean age* = 75.19 months, *SD* = 3.58) and 215 boys (*mean age* = 74.94 months, *SD* = 3.75) and two other children with missing gender information.

### Procedure

In August, the volunteer teachers were trained for the three-tiered group assessment procedure, which was conducted in September, January, and May during the preprimary education year. The following number skill components were assessed: symbolic number comparison (September, January, and May), mapping (September, January, and May), and verbal counting skills (January and May). The tools were piloted before the actual study to ensure that the participating teachers were able to follow the instructions for the assessment procedure, and that the items would measure expected dimensions and cover individuality of skill-levels. The teachers then administered the assessment procedure within their own preprimary education group during small group sessions (of 5-8 children). The teachers instructed the tasks item by item to the children who responded by cross marking one of the three alternatives presented on the paper. After each assessment point, the teachers returned all materials for each assessment point to the research assistants who were trained to work with the data. This procedure was carried out at each assessment time point (September, January, and May). After each assessment point, we tested the validity and difficulty of items. Based on the results and expected skills development, the amount of items were reduced and changed and new skill components added to the following screener for receiving meaningful variance.

With individual and small group assessment settings (attention), and with permission to repeat the instructions (working memory), as well as by using multiple-choice, paper-and-pencil items without time limits (response inhibition), the demand for executive functioning skills during the assessments was thought to be diminished. By varying and challenging the number skill components over time (i.e., changing numerical distances among alternatives, growing the number area, and adding assessed components), the difficulty level was thought to increase from fall to winter to spring. With this decision, practitioners could screen weaknesses at different cross-sectional time points by comparing the individual performance levels to typically developing children with diminished risk of a potential floor or ceiling effect or a test–retest effect.

### Measures

#### Symbolic Number Comparison

Symbolic number comparison skill was assessed at time point 1 (eight items, Cronbach’s alpha = 0.88) and time point 2 (six items, Cronbach’s alpha = 0.91) in small group settings. At both time points, the first half of the assessment tasks included items from which the child was asked to choose and mark the largest written number among three alternatives, presented horizontally (e.g., 9, 4, and 7). The second half consisted of tasks in which the child was asked to choose the smallest written number (e.g., 6, 10, and 8). Each item was coded as zero (incorrect) or 1 point (correct) or as an empty cell (missing value), so that the approximate number comparison formed a categorical variable for the analysis. At time point 3, the number comparison task required exact comparison skill (four items, Cronbach’s alpha = 0.59). The child was asked to choose which of the three alternatives included one more, two more, one fewer, and two fewer than the item originally presented. Each item was coded as zero (incorrect) or 1 point (correct) or as an empty cell (missing value) and was set as categorical items for the analysis to first evaluate their validity and difficulty level in assessing number comparison skills. Based on the item difficulty analysis (see section “Data Analysis” and the Appendix), three NC variables, one per time point, were included in the final analysis as parceled variables (NC_1, NC_2, and NC_3).

#### Mapping

Mapping skills were assessed at time point 1 (16 items, Cronbach’s alpha = 0.88) and time point 2 (eight items, Cronbach’s alpha = 0.71) in small group settings. The test included four types of tasks each consisting of four items (time point 1) or two items (time point 2). For each task type, the child was asked to choose the corresponding numerical representation from among three alternatives. First, number words were contrasted with quantities (dots), and then number words were contrasted with written number symbols (e.g., the number word “eight” was said aloud and the written symbols 7, 9, and 8 were presented), then, quantities were contrasted with written symbols (without verbal hints), and finally, written symbols were contrasted with quantities. Each item was coded as zero (incorrect) or 1 point (correct) or as an empty cell (missing value) so that cardinal number concept skill formed a categorical variable for the analysis. At time point 3, the task consisted only of four items (Cronbach’s alpha = 0.60) in which the child was asked to mark the 12th, the 17th, every 2nd, and finally, every 3rd item among several alternatives presented horizontally for each task. Each item was coded as zero (incorrect) or 1 point (correct) or as an empty cell (missing value) and was set as categorical items for the analysis to first evaluate their validity and difficulty level in assessing mapping skills. Based on item difficulty analysis (see section “Data Analysis” and the Appendix), three MS variables, one per time point, were included in the final analysis as parceled variables (MS_1, MS_2, and MS_3).

#### Verbal Counting

Verbal counting was assessed individually at time point 2 and at time point 3 with identical tasks (nine items, Cronbach’s alpha = 0.84 and 0.82, respectively). First, the child was asked to count forward starting from one. This task was divided into three subtasks: to count correctly up to the number word 10, to the number word 20, and to the number word 30. Second, the child was asked to count backward, again in three subtasks: to count backward correctly from 5 to 1, from 12 to 8, and from 20 to 16. Third, the child was asked to skip count by twos, again in three subtasks: to count correctly up to the number word 10, to the number word 18, and to the number word 30. Each item was coded as zero (incorrect) or 1 point (correct) or as an empty cell (missing value) and was set as categorical items for the analysis to first evaluate their validity and difficulty level in assessing verbal counting skills. Based on item difficulty analysis (see section “Data Analysis” and the Appendix), two VC variables, one per time point, were included in the final analysis as parceled variables (VC_2 and VC_3; numbers indicating the time point).

#### Basic Arithmetic Story Problems

Basic arithmetic was assessed at time point 3 (eight items, Cronbach’s alpha = 0.63) in small group settings. Four of the assessment tasks were verbally presented addition tasks (*A boy has three fishes. He gets two more fishes. How many fishes does he have now?*), in which the children needed to give their responses by marking the correct number symbol among three alternatives presented horizontally. With a similar procedure, the child was asked to respond to four other tasks that were subtraction tasks (*A girl has five keys. She gives two keys away. How many keys does she have now?*). Each item was coded as zero (incorrect) or 1 point (correct) or as an empty cell (missing value). Because this study focused on the prerequisite skills for arithmetic (NC, MS, and VC), this task was included in *post hoc* analysis only as a sum score of eight items for testing potential differences in basic arithmetic between hypothetically meaningful profile groups.

### Data Analysis

First, item response theory (IRT) analysis was needed and conducted for each of the eight number skill components: to assess the items’ ability to measure the dimensions and cover all the individuals’ skills level, to evaluate item difficulties, and factor loadings. Model parameters were estimated using the weighted least squares means and variance adjusted (WMLSV estimator) estimation in Mplus version 7.11 (Muthén and Muthén, 1998-2012). Goodness-of-fit was evaluated based on the following criteria: chi-square test of model fit (χ^2^), root-mean-square error of approximation (RMSEA), comparative fit index (CFI), Tucker-Lewis index (TLI), and weighted root-mean-square residual (WRMR). Values for well-fitting measurement models were as follows: RMSEA < 0.06, CFI > 0.95, TLI > 0.95, and WRMR < 0.09. To reduce the number of estimated parameters for the sample size, parcels were formed using item difficulty information from the IRT analysis. The classification of individual items into the parcels was also based on content (e.g., different types of verbal counting items, including counting on, counting backward from a given number, and counting on by twos, were mixed in each verbal counting parcel to add balance among the three parcels). The goodness-of-fit with the estimates RMSEA, TLI, CFI, and WRMR for different types of latent number skill components are presented in the Appendix along the item difficulty information and factor loadings per dimension (NC_1, MS_1, and VC_2). The time points, when the components were assessed the first time, were used because the following components were formed from the originally presented items (i.e., the following assessment points contained an equal or smaller number of items compared to previous assessment points). To better evaluate the validity of the NC and MS components at time point 3 (because the reported Cronbach alpha values were relatively small probably due to the small number of items, 0.59 and 0.60, respectively), the factor loadings for these dimensions (NC_3 and MC_3) are also presented separately in the Appendix. Correlations between the eight latent number skill factors are presented in **Table 1** for the whole sample (*N* = 440). Based on the measurement models, factor scores were computed for use in the second step of the analysis. Item difficulty, standardized factor loadings of each item, and the parceling information are presented in the Appendix.

**Table 1:** Correlations between early number skill component factor scores.

Factor	1	2	3	4	5	6	7
(1) Number Comparison (NC_1)							
(2) Mapping Skills (MS_1)	0.695						
(3) Number Comparison (NS_2)	0.723	0.637					
(4) Mapping Skills (MS_2)	0.533	0.785	0.604				
(5) Verbal Counting (VC_2)	0.556	0.710	0.706	0.616			
(6) Number Comparison (NC_3)	0.616	0.713	0.715	0.677	0.725		
(7) Mapping Skills (MS_3)	0.535	0.684	0.764	0.619	0.639	0.764	
(8) Verbal Counting (VC_3)	0.516	0.710	0.665	0.612	0.907	0.733	0.684

Second, LPA across a total of eight latent number component factor scores was used to empirically identify potential skill profile groups (RQ1). Mplus provides several statistical fit indices for deciding the number of latent classes. In the present study, individuals (*N* = 440) were classified into different latent profile groups using the following criteria: the Akaike information criterion (AIC), the Bayesian information criterion (BIC), the adjusted BIC, the entropy index, average posterior probabilities, and statistical test results for the Lo-Mendell-Rubin Likelihood ratio test (LMRL), Lo-Mendell-Rubin test (LMR), and bootstrap likelihood ratio test (BLRT). As the three screening tools were developed to differentiate the potential skill levels of poorly performing children and their potential differences on separate number skill components (RQ2), LPA was terminated when the average posterior probabilities and class counts proposed new groups of near-average- and/or high-average-performing children with small class counts. Analyses for between-profile-group differences in terms of preprimary education group, age, and gender (RQ3) were conducted using the auxiliary option in Mplus (Muthén and Muthén, 1998-2012). Finally, for testing potential group differences in BA, the independent samples *t*-test was used (RQ4).

## Results

### Research Question 1

**FIGURE 2:**
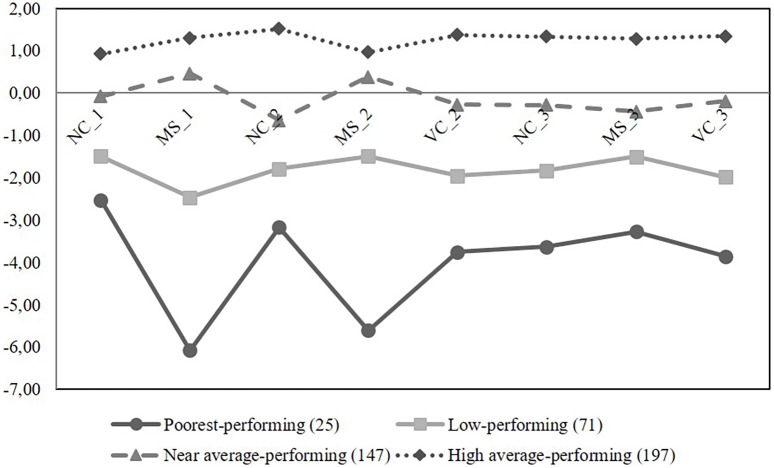
Latent profile groups across three time points with standardized estimates for intercept of each latent factor. COMP(a), approximate number comparison skill; NC(c), cardinal number concept skill; VC, verbal counting skill; COMP(e), exact number comparison skill; NC(o), ordinal number concept skill; 1–3, time points 1–3.

In LPA, the parsimonious number of classes was four with class counts of 25 (the poorest-performing; 6%), 71 (low-performing; 16%), 147 (near-average-performing; 33%), and 197 (high-average-performing; 45%) when all eight latent basic number skill components were included in the analysis (**Figure 2**). Average latent class probabilities for most likely latent class membership were 0.999 for the poorest-performing group, 0.964 for the low-performing group, 0.954 for the near-average-performing group, and 0.970 for the high-average-performing group indicating very high stability of group membership. Model fit indices for different class solutions are presented in **Table 2**.

**Table 2:** Standardized fit indices for latent profile analysis over early number skill components.

No. of classes	LL	No. of free parameters	AIC	BIC	Adj. BIC	Entropy	VLMR	Adj. VLMR	BLRT
2	−4040.83	25	8131.65	8233.82	8154.49	0.96	*p* < 0.001	*p* < 0.001	*p* < 0.001
3	−3661.77	34	7391.55	7530.50	7422.60	0.93	*p* = 0.054	*p* = 0.056	*p* < 0.001
**4**	−**3429.83**	**43**	**6945.66**	**7121.39**	**6984.93**	**0.94**	***p* < 0.001**	***p* < 0.001**	***p* < 0.001**
5	−3313.62	52	6731.25	6943.76	6778.74	0.94	*p* = 0.085	*p* = 0.089	*p* < 0.001
6	−3238.58	61	6599.16	6848.45	6654.87	0.95	*p* = 0.225	*p* = 0.230	*p* < 0.001

*LL, log-likelihood; AIC, Akaike Information Criterion; BIC, Bayesian Information Criterion; Adj., adjusted; VLMR, Vuong-Lo-Mendell-Rubin test; BLRT, Bootstrap Likelihood-Ratio-Test. The best-fitting solution is shown in boldface.*

### Research Question 2

Based on confidence interval comparisons, all four profile groups differed statistically significantly from each other on every latent skill component over the preprimary education year (**Table 3**). Further, the poorest-performing children performed equally poorly in number comparison and mapping tasks while for the other groups of children mapping task seemed to somewhat be easier than number comparison task. The percentages of accuracy were 35% for the poorest-, 51% for the low-, 79% for the near-average-, and 97% for the high-average-performing children in number comparison task. The respective percentages were 38, 69, 93, and 98% for the group of poorest-, low-, near-average-, and high-average-performing children in mapping. That is why mapping skill seemed to most differentiate the poorest-performing children from the other profile groups (**Figure 2**). In more detail, the items that required mapping between quantities and written number symbols and vice versa were the most difficult for the poorest-performing children. The percentages of correctly mapped numerical representations in the poorest-performing group were as follows: 23% for quantities to number symbols and vice versa and 54% for number words to quantities and vice versa. The respective percentages were 56 and 81% for the group of low-performing children; 90 and 96% for the near-average-; and 97 and 99% for the high-average-performing children.

**Table 3:** Standardized estimates for intercepts with confidence intervals in four-class solution over early number skill components.

	Latent group											
	Poorest-performing (*25*)			Low-performing (*71*)			Near average-performing (*147*)			High average-performing (*197*)		
Latent variable	*i (SE)*	*CI (99%)*		*i (SE)*	*CI (99%)*		*i (SE)*	*CI (99%)*		*i (SE)*	*CI (99%)*	
		Lower	Upper		Lower	Upper		Lower	Upper		Lower	Upper
Time point 1												
Number comparison	−2.53 (0.27)	−2.15	−1.351	−1.49 (0.20)	−1.349	−0.71	−0.08 (0.12)	−0.28	0.17	0.92 (0.06)	0.57	0.69
Mapping skills	−6.08 (0.54)	−3.17	−2.33	−2.46 (0.26)	−1.40	−0.83	0.46 (0.11)	0.09	0.32	1.30 (0.10)	0.55	0.63
Time point 2												
Number comparison	−3.17 (0.29)	−2.12	−1.40	−1.79 (0.16)	−1.21	−0.78	−0.64 (0.12)	−0.55	−0.17	1.52 (0.10)	0.76	0.93
Mapping skills	−5.61 (0.56)	−3.56	−2.36	−1.49 (0.25)	−1.10	−0.47	0.38 (0.08)	0.11	0.30	0.96 (0.08)	0.45	0.56
Verbal counting	−3.75 (0.32)	−2.38	−1.77	−1.95 (0.21)	−1.32	−0.84	−0.27 (0.14)	−0.34	0.05	1.37 (0.11)	0.67	0.84
Time point 3												
Number comparison	−3.63 (0.33)	−2.44	−1.69	−1.83 (0.18)	−1.27	−0.81	−0.29 (0.10)	−0.32	−0.01	1.33 (0.12)	0.64	0.87
Mapping skills	−3.27 (0.32)	−2.40	−1.55	−1.50 (0.13)	−1.09	−0.71	−0.44 (0.10)	−0.41	−0.11	1.28 (0.11)	0.64	0.90
Verbal counting	−3.86 (0.34)	−2.47	−1.79	−1.98 (0.20)	−1.34	−0.85	−0.19 (0.13)	−0.30	0.09	1.34 (0.09)	0.67	0.81

*I, intercept; CIs, confidence intervals.*

### Research Question 3

There were no between-group differences in terms of participating in preeducation instruction in a certain kindergarten group (*n* = 30). However, according to the chi-square test with basic precursors, the high-average-performing children were statistically significantly older (mean age, 75.84 months, *SE* = 0.25) than the children in the three other groups (the poorest-performing *_mean_* = 73.87 months, *SE* = 0.78, chi-square = 5.90, *p* = 0.015; low-performing _mean_ = 74.04 months, *SE* = 0.48, chi-square = 11.28, *p* = 0.001; near-average-performing _mean_ = 74.69 months, *SE* = 0.31, chi-square = 7.97, *p* = 0.005). Finally, there appeared to be more boys within the poorest-performing group than in the near- (chi-square = 6.85, *p* = 0.009) or high-average-performing (chi-square = 7.18, *p* = 0.007) groups but not compared to the low-performing group. To conclude, the poorest- and low-performing profile groups did not differ in terms of kindergarten group, age, or gender.

### Research Question 4

Latent profile analysis method showed that the poorest- and low-performing profile groups were unique. To confirm the result, an independent-samples *t*-test was used to examine the potential group difference in basic arithmetic. According to the *t*-test, the poorest-performing children performed statistically significantly poorer in basic arithmetic than the low-performing children (the poorest-performing *_mean_* = 4.61, *SD* = 1.67; low-performing _mean_ = 5.89, *SD* = 1.70; *t*(92) = −3.14, *p* = 0.002, *d* = 0.45).

## Discussion

In the present study, latent profile analysis was used to identify potential performance-level groups among 440 Finnish children (6- to 7-year-olds) with distinct number skill profiles. The performance levels in three number skill components, with which fluent arithmetic skills have typically been predicted, were assessed three times during the preprimary education semester in September, January, and May. The components were number comparison, mapping between different numerical representations (quantities, number words, and number symbols), and verbal counting.

The results of the present study revealed four types of performance profile groups across number comparison, mapping, and verbal counting skills. There was a statistically significant difference in all number skill components between the poorest- (6%), low- (16%), near-average- (33%), and high-average-performing children (45%). Based on these results, the poorest- and low-performing children seem to need acute support for all early number skill components. In particular, the poorest-performing children seem to need specific training for number comparison and mapping skills. Especially, the task types that required exact mapping of quantities with number symbols, as well as number symbols with quantities were the most difficult for the poorest-performing children. Instead, the percentages of accuracy in tasks dealing with number words (number word–quantity and number word–number symbol mapping) were higher. Moreover, the poorest-performing children differed statistically significantly from low-performing children in basic addition and subtraction story problem-solving skills (*d* = 0.45). The poor performance in the early number and story problem-solving skills indicate a clear risk for arithmetical difficulties especially among the poorest-performing children.

Aligned with previous literature, the LPA in the present study suggests that 96 children (22% of the total sample) performed less well than the near- or high-average-performing children. Of these 96 children, 71 formed one unique profile group (low-performing children, representing approximately 16% of the total sample), and 25 formed another unique profile group (the poorest-performing children, representing approximately 6% of the total sample) with high stability of group membership. These proportions (16 and 6%) seem somewhat to be in line with previous findings that children struggling with math skills could have different types of growth rates if their initial performance level varies between the 11th and 25th percentiles or falls below the 10th percentile (Murphy et al., 2007; Morgan et al., 2009, 2011; Salaschek et al., 2014). This study offers support for this phenomenon by showing that these performance-level differences already exist before formal schooling. The findings are also in line with previous literature (concerning performance levels) although the present study used only very basic number skill components instead of school mathematics (Murphy et al., 2007; Morgan et al., 2009, 2011; Salaschek et al., 2014). Finally, interestingly, the proportion of the poorest-performing children (6%) found in this study was comparable to the estimated prevalence of children with MD who are typically diagnosed as having deficits in arithmetic fluency at older age levels (varying between at 3–7, 5–7, and 5–8%; Landerl and Moll, 2010; Butterworth et al., 2011; Geary, 2011).

The findings also suggest that the poorest-performing children have serious deficits in all early number skills. Further, the percentages of correctness were at the same level within the poorest-performing children in number comparison (35) and in mapping (38). However, the other groups seemed to perform better in mapping than in number comparison task. The percentages of correctness were 69, 93, and 98 within the low-, near-average-, and high-average-performing children, respectively. In number comparison, the corresponding percentages within the low-, near-average-, and high-average-performing children were 51, 79, and 97, respectively. That is why the mapping task differentiated the poorest-performing children from the other groups the most.

In more detail, in mapping task, the poorest-performing children seemed to have more serious deficits than low-performing children especially in matching written number symbols to the corresponding quantities and vice versa. The poorest-performing children showed less serious deficits when verbal number words were included in the mapping tasks. It follows that these findings cannot be explained (at least not fully) by weak dot counting skills or by verbal deficits, as a comparable performance in that case would have been found in written symbol–quantity and verbal number word–quantity mapping tasks. Moreover, the number word–written symbol mapping task was easier for the poorest-performing children than the written symbol–quantity task. This finding lends further support to the suggestion that the most serious deficits are in finding associations between written number symbols and quantities and thus, support the theoretical hypothesis of children with MD having deficits in accessing numerical meaning from written number symbols (De Smedt and Gilmore, 2011). This was supported also by the fact that tasks dealing with number words were easier for the poorest-performing children. That is why number sense (or module) deficit was not supported in our study.

From the developmental perspective, these findings are in line with previous studies suggesting stable and even increasing differences between the unique poorest- and low-performing profile trends (Murphy et al., 2007; Morgan et al., 2009, 2011; Geary, 2011; Wong et al., 2014). Additionally, the mapping tasks operated with number words are developmentally more familiar to children at first than the tasks requiring understanding of the direct quantity–symbol relationship without verbal support (Dehaene, 1992; von Aster and Shalev, 2007; Geary, 2013). To link these findings to longitudinal studies focusing approximately on the same age level, this study showed that children’s age is positively associated with performance level as was shown in Jordan et al. (2006) longitudinal study. Older children may have more experience with numbers and (numerical) language than their younger age peers. Therefore, the differences in readiness to benefit from early instructions and participate in peer discussions can be greater between the age levels at the beginning of formal schooling. In contrast to previous findings (for a review, Jordan et al., 2006, 2007; Devine et al., 2013), boys were overrepresented among the low-performing children in the present study in comparison to near- and high-average-performing children, but the poorest- and low-performing groups did not differ by age or gender. The contradictory findings concerning gender differences in mathematics might be due to the methods used for testing differences (Devine et al., 2013). In general, in population-based studies, there are no clear gender differences in the mean level (for a review, see Hyde et al., 2008; Lindberg et al., 2010), but a difference can be found among lower- or higher-performing children (Devine et al., 2013; Stoet and Geary, 2013).

### Implications for Educational Practice

The present findings suggest that theoretically valid screening tools have potential to identify children in need of extra support in early number skill components. Moreover, by assessing number comparison, mapping, and verbal counting, it is possible to identify a subgroup of children, with a corresponding prevalence rate of MD, whose poor number skill performance seems to be stable during the whole preprimary education year. The findings suggest that educational practices for early identification of MD risk and early number skills intervention should focus on the most basic skills, especially on quantity-number symbol mapping skill (and vice versa) which most differentiates the poorest-performing children from low-performing children. The stability of poor performance levels found throughout preprimary education indicates a need for systematic progress monitoring of number skill development, as well as planning and offering appropriate mathematical support at the very beginning of formal schooling or perhaps earlier.

### Limitations

Deficits in working memory, language, and visuospatial skills (Raghubar et al., 2010; Geary, 2011), processing speed (Willcutt et al., 2013), and certain domains of executive functioning (Friso-van den Bos et al., 2013; Price and Fuchs, 2016; Price and Wilkey, 2017) are also found to be associated with arithmetic skills or math performance more generally. Thus, in future studies, by controlling for general domain skills (see also Kaufmann et al., 2013) and task-specific requirements (De Smedt et al., 2013; Price and Wilkey, 2017), we could better understand the potential qualitative differences between the poorest-performing and low-performing children (Geary, 2011). We also could better identify those children (most) at risk for MD and likewise, plan meaningfully targeted support for individual needs (Landerl et al., 2009; Rubinsten and Henik, 2009; Butterworth et al., 2011). Unfortunately, in our study administered by teachers, we could not measure these general cognitive skills. We only tried to minimize the demand of executive functioning skills by using a certain type of assessment procedure (e.g., small group sessions, permission to repeat the instructions, only a cross-marking requirement in responses, and non-speeded tasks).

The study tools were developed and tested for practical use. The aim was to develop a set of screeners that could first identify (alert) children in need of extra evaluation and immediate early number skill support (at the beginning of pre-primary education) and then evaluate the progress (in winter and spring times). For this reason, a larger amount of basic skills’ items were included into the first screener and then the amount of these items were reduced for being able to add theoretically and developmentally meaningful skills’ items into the following screeners (winter and spring) without increasing the assessment effort. This causes three clear limitations for this study. First, reducing the number of items and by changing the assessed skill components we were not able to analyze the number skill development comprehensively (LPA was used instead of growth curve models). Second, by reducing the number of items, some of the sub-skill dimensions showed low reliability values although the reliability for the three screeners as a whole were relatively high (Cronbach alpha values being 0.91; 0.88; and 0.84 respectively). That is why IRT- and factor analysis were conducted for showing the validity of skill components. Third, we were not able to measure all important skills related to arithmetic development. For instance, number line estimation task (as one of the critical measures) would require careful interpretations of the correctness and would therefore be difficult to conduct in screeners meant for practical use. Further, to assess non-symbolic comparison skills with a paper-and-pencil task (which would have been the case in our study), well-controlled items would have been needed (controlling for instance for area, ratio, distance, and response time). However, as they are important, both skills could be individually assessed for example after a classroom-based screening situation for confirming the skill-levels.

One main criticism of using LPA is that the proposed number of classes may not refer to existing subpopulations within the population (Bauer and Curran, 2004). However, in this study, the best-fitting solution (four profile groups) and the alternative solutions (five or six profile groups) proposed one clear group of the poorest-performing children, in which the latent early number skill components differ most from the other skill-level groups. Thus, findings concerning the poorest-performing children seemed reliable.

## Ethics Statement

This study was carried out in accordance with the recommendations of the Finnish Advisory Board on Research Integrity. The protocol was approved by the Niilo Mäki Institute. All subjects gave written informed consent in accordance with the Finnish Advisory Board on Research Integrity.

## Author Contributions

JS main work of the whole paper. TK contribution to context. AT contribution to methods.

## Conflict of Interest Statement

The authors declare that the research was conducted in the absence of any commercial or financial relationships that could be construed as a potential conflict of interest.
