# Supplementary material for: Individuality in the Early Number Skill Components Underlying Basic Arithmetic Skills
Source: Front Psychol. 2018 Jul 2;9:1056. doi: 10.3389/fpsyg.2018.01056 (PMC6036168; doi:10.3389/fpsyg.2018.01056)
Supplement: Supplementary file 1 [file Table_1.DOCX]

Table 1

*Correlations Between Early Number Skill Component Factor Scores*

| Factor | 1 | 2 | 3 | 4 | 5 | 6 | 7 |
| --- | --- | --- | --- | --- | --- | --- | --- |
| 1. Number Comparison (NC_1) |  |  |  |  |  |  |  |
| 2. Mapping Skills (MS_1) | .695 |  |  |  |  |  |  |
| 3. Number Comparison (NS_2) | .723 | .637 |  |  |  |  |  |
| 4. Mapping Skills (MS_2) | .533 | .785 | .604 |  |  |  |  |
| 5. Verbal Counting (VC_2) | .556 | .710 | .706 | .616 |  |  |  |
| 6. Number Comparison (NC_3) | .616 | .713 | .715 | .677 | .725 |  |  |
| 7. Mapping Skills (MS_3) | .535 | .684 | .764 | .619 | .639 | .764 |  |
| 8. Verbal Counting (VC_3) | .516 | .710 | .665 | .612 | .907 | .733 | .684 |
