# Supplementary material for: Individuality in the Early Number Skill Components Underlying Basic Arithmetic Skills
Source: Front Psychol. 2018 Jul 2;9:1056. doi: 10.3389/fpsyg.2018.01056 (PMC6036168; doi:10.3389/fpsyg.2018.01056)
Supplement: Supplementary file 2 [file Table_2.DOCX]

Table 2

*Standardized Fit Indices for Latent Profile Analysis over Early Number Skill Components*

| No. of classes | LL | No. of free parameters | AIC | BIC | Adj. BIC | Entropy | VLMR | Adj. VLMR | BLRT |
| --- | --- | --- | --- | --- | --- | --- | --- | --- | --- |
| 2 | -4040.83 | 25 | 8131.65 | 8233.82 | 8154.49 | 0.96 | *p* < .001 | *p* < .001 | *p* < .001 |
| 3 | -3661.77 | 34 | 7391.55 | 7530.50 | 7422.60 | 0.93 | *p* = .054 | *p* = .056 | *p* < .001 |
| **4** | **-3429.83** | **43** | **6945.66** | **7121.39** | **6984.93** | **0.94** | ***p* < .001** | ***p* < .001** | ***p* < .001** |
| 5 | -3313.62 | 52 | 6731.25 | 6943.76 | 6778.74 | 0.94 | *p* = .085 | *p* = .089 | *p* < .001 |
| 6 | -3238.58 | 61 | 6599.16 | 6848.45 | 6654.87 | 0.95 | *p* = .225 | *p* = .230 | *p* < .001 |

### *Note.* LL = log-likelihood; AIC = Akaike Information Criterion; BIC = Bayesian Information Criterion; Adj. = Adjusted; VLMR = Vuong-Lo-Mendell-Rubin test;

### BLRT = Bootstrap Likelihood-Ratio-Test. The best-fitting solution is shown in boldface.
