# Supplementary material for: Individuality in the Early Number Skill Components Underlying Basic Arithmetic Skills
Source: Front Psychol. 2018 Jul 2;9:1056. doi: 10.3389/fpsyg.2018.01056 (PMC6036168; doi:10.3389/fpsyg.2018.01056)
Supplement: Supplementary file 3 [file Table_3.DOCX]

Table 3

*Standardized Estimates for Intercepts with Confidence Intervals in Four-Class Solution over Early Number Skill Components*

|  | Latent group | | | | | | | | | | | |
| --- | --- | --- | --- | --- | --- | --- | --- | --- | --- | --- | --- | --- |
|  | Poorest-performing (*25*) | | | Low-performing (*71*) | | | Near average-performing (*147*) | | | High average-performing (*197*) | | |
| Latent variable | *i (S.E.)* | *CI (99 %)* | | *i (S.E.)* | *CI (99 %)* | | *i (S.E.)* | *CI (99 %)* | | *i (S.E.)* | *CI (99 %)* | |
|  |  | Lower | Upper |  | Lower | Upper |  | Lower | Upper |  | Lower | Upper |
| Time point 1 |  |  |  |  |  |  |  |  |  |  |  |  |
| Number Comparison | -2.53 (0.27) | -2.15 | -1.351 | -1.49 (0.20) | -1.349 | -0.71 | -0.08 (0.12) | -0.28 | 0.17 | 0.92 (0.06) | 0.57 | 0.69 |
| Mapping Skills | -6.08 (0.54) | -3.17 | -2.33 | -2.46 (0.26) | -1.40 | -0.83 | 0.46 (0.11) | 0.09 | 0.32 | 1.30 (0.10) | 0.55 | 0.63 |
|  |  |  |  |  |  |  |  |  |  |  |  |  |
| Time point 2 |  |  |  |  |  |  |  |  |  |  |  |  |
| Number Comparison | -3.17 (0.29) | -2.12 | -1.40 | -1.79 (0.16) | -1.21 | -0.78 | -0.64 (0.12) | -0.55 | -0.17 | 1.52 (0.10) | 0.76 | 0.93 |
| Mapping Skills | -5.61 (0.56) | -3.56 | -2.36 | -1.49 (0.25) | -1.10 | -0.47 | 0.38 (0.08) | 0.11 | 0.30 | 0.96 (0.08) | 0.45 | 0.56 |
| Verbal counting | -3.75 (0.32) | -2.38 | -1.77 | -1.95 (0.21) | -1.32 | -0.84 | -0.27 (0.14) | -0.34 | 0.05 | 1.37 (0.11) | 0.67 | 0.84 |
|  |  |  |  |  |  |  |  |  |  |  |  |  |
| Time point 3 |  |  |  |  |  |  |  |  |  |  |  |  |
| Number Comparison | -3.63 (0.33) | -2.44 | -1.69 | -1.83 (0.18) | -1.27 | -0.81 | -0.29 (0.10) | -0.32 | -0.01 | 1.33 (0.12) | 0.64 | 0.87 |
| Mapping Skills | -3.27 (0.32) | -2.40 | -1.55 | -1.50 (0.13) | -1.09 | -0.71 | -0.44 (0.10) | -0.41 | -0.11 | 1.28 (0.11) | 0.64 | 0.90 |
| Verbal Counting | -3.86 (0.34) | -2.47 | -1.79 | -1.98 (0.20) | -1.34 | -0.85 | -0.19 (0.13) | -0.30 | 0.09 | 1.34 (0.09) | 0.67 | 0.81 |

Notes. I = Intercept; CI = Confidence intervals.
