# Supplementary material for: Individuality in the Early Number Skill Components Underlying Basic Arithmetic Skills
Source: Front Psychol. 2018 Jul 2;9:1056. doi: 10.3389/fpsyg.2018.01056 (PMC6036168; doi:10.3389/fpsyg.2018.01056)
Supplement: Supplementary file 4 [file Data_Sheet_1.DOCX]

Appendix

*Fit indices with factor loadings, item difficulty and parcel information of studied skill components*

|  | Skill Component | RMSEA | CFI | TLI | WRMR | Loading | Difficulty | Parcel |
| --- | --- | --- | --- | --- | --- | --- | --- | --- |
| Number Comparison (NC_1) | | .000 | 1.000 | 1.000 | .453 |  |  |  |
|  | item number 1 |  |  |  |  | 0.881 | -0.446 | 2 |
|  | 2 |  |  |  |  | 0.946 | -0.717 | 1 |
|  | 3 |  |  |  |  | 0.828 | -1.360 | 3 |
|  | 4 |  |  |  |  | 0.949 | -0.867 | 1 |
|  | 5 |  |  |  |  | 0.843 | -1.237 | 3 |
|  | 6 |  |  |  |  | 0.844 | -1.004 | 1 |
|  | 7 |  |  |  |  | 0.718 | -1.746 | 2 |
|  | 8 |  |  |  |  | 0.917 | -0.951 | 2 |
| Mapping Skills (MS_1) | | .039 | .986 | .983 | .940 |  |  |  |
|  | item number 1 |  |  |  |  | 0.831 | -1.790 | 1 |
|  | 2 |  |  |  |  | 0.648 | -1.557 | 3 |
|  | 3 |  |  |  |  | 0.693 | -1.658 | 1 |
|  | 4 |  |  |  |  | 0.790 | -0.548 | 2 |
|  | 5 |  |  |  |  | 0.941 | -1.333 | 2 |
|  | 6 |  |  |  |  | 0.951 | -1.196 | 1 |
|  | 7 |  |  |  |  | 0.920 | -1.073 | 2 |
|  | 8 |  |  |  |  | 0.874 | -0.871 | 1 |
|  | 9 |  |  |  |  | 0.618 | -2.115 | 2 |
|  | 10^a^ |  |  |  |  | 0.723 | -2.160 | 1 |
|  | 11^a^ |  |  |  |  | 0.627 | -2.489 | 3 |
|  | 12 |  |  |  |  | 0.707 | -1.491 | 2 |
|  | 13^a^ |  |  |  |  | 0.763 | -2.215 | 3 |
|  | 14^a^ |  |  |  |  | 0.817 | -1.722 | 2 |
|  | 15 |  |  |  |  | 0.602 | -2.693 | 3 |
|  | 16 |  |  |  |  | 0.865 | -1.740 | 3 |
| Verbal Counting (VC_2) | | .031 | .998 | .997 | 0.790 |  |  |  |
|  | item number 1 |  |  |  |  | .366 | -7.078 | 2 |
|  | 2 |  |  |  |  | .937 | -1.835 | 1 |
|  | 3 |  |  |  |  | .872 | -1.004 | 1 |
|  | 4 |  |  |  |  | .803 | -2.209 | 3 |
|  | 5^c^ |  |  |  |  | .845 | -1.123 | 3 |
|  | 6^c^ |  |  |  |  | .856 | -0.450 | 2 |
|  | 7^c^ |  |  |  |  | .809 | -0.999 | 2 |
|  | 8^c^ |  |  |  |  | .801 | -0.386 | 1 |
|  | 9^c^ |  |  |  |  | .866 | 0.000 | 3 |
| Number Comparison (NC_3) | | .042 | .994 | .983 | .465 |  |  |  |
|  | item number 1 |  |  |  |  | .349 | -3.940 |  |
|  | 2 |  |  |  |  | .813 | -1.103 |  |
|  | 3 |  |  |  |  | .764 | -1.675 |  |
|  | 4 |  |  |  |  | .848 | -0.923 |  |
| Mapping Skills (MS_3) | | .000 | 1.000 | 1.018 | .089 |  |  |  |
|  | item number 1 |  |  |  |  | .770 | -1.295 |  |
|  | 2 |  |  |  |  | .765 | -1.008 |  |
|  | 3^b^ |  |  |  |  | .589 | -1.026 |  |
|  | 4^b^ |  |  |  |  | .413 | -0.614 |  |

*Note.* RMSEA = Root mean square error of approximation; CFI = Comparative fit index; TLI = Tucker Lewis Index; WRMR = Weighted Root Mean Square Residual; Loadings = Standardized factor loadings; Difficulty = Theta difficulties based on item response theory (IRT) parametrization; Parcel = Parcel number describes how items were set into parcels if the number of sub-skill items was more than six.

^a^Freeing the residual correlations (10 with 11 = .937; 13 with 14 = .792).

^b^Freeing the residual correlation (3 with 4 = .554).

^c^Freeing the residual correlations (5 with 6 = .684; 7 with 8 = .729; 8 with 9 = .871).
